# Exploring a new model of cell wall regulation: identification and expression of two putative SHINEs transcription factors in *Eucalyptus*

**DOI:** 10.1186/1753-6561-5-S7-P166

**Published:** 2011-09-13

**Authors:** Wesley L Marques, Marcela Salazar, Eduardo Camargo, Jorge Lepikson-Neto, Danieli Cristina Gonçalves, Leandro Costa do Nascimento, Carla Garcia, Adriano Almeida, Gonçalo Pereira

**Affiliations:** 1 State University of Campinas – UNICAMP, Brazil; 2 International Paper

## Background

*Eucalyptus* forests are a competitive and efficient alternative to convert carbon from the atmosphere in cellulose, an important source for paper manufacture and bioenergy production.

To obtain transgenic *Eucalyptus* with important traits improved it is necessary to make modifications in genes that affect the final phenotype. One interesting gene that follows this requisite was recently found: this is the *AtSHN2* gene (*Arabidopsis thaliana SHINE 2*).

*AtSHN2* codifies to a Transcription Factor known as “Arabidopsis *SHINE/WAX INDUCER*”. Instead of inducing drought tolerance in transgenic rice (*Oryza sativa*), *AtSHN2* overexpression causes: i) 34% increase in the cellulose content; ii) 45% reduction in lignin content and iii) increase in wood digestibly (elevated S:G ratio) with no compromise in plant strength and performance [1].

The discovery of *AtSHN2* function in plant cell wall formation, led Ambavaram and collaborators [1] to perform other studies and ultimately to propose the following model: *AtSHN2* regulates positively *MYB* transcription factors (TF) related to cellulose synthesis and it down-regulates *MYBTF’s* related to lignin formation. At the same time, *SHINE* can repress *NAC TFthat controls MYB expression*[*1*]*.*

As a consequence of the interesting phenotype achieved through *AtSHN2* overexpression in rice, this work focused on the identification and analyses of *AtSHN* orthologues in *Eucalyptus*. Bioinformatics tools were used to search for *AtSHN* similar genes in *Eucalyptus.* Moreover, the expression profile of the corresponding genes in *Eucalyptus* was evaluated to prove their role as *AtSHN*. To carry it on, the expression experiments were done with flower, leaf and xylem. If the *Eucalyptus* putative*SHINE's* has the same function of the *AtSHN’s*,, gene expression in flower tissues will be the highest [2]. This is because it is known that *AtSHN’s* genes are preferentially expressed in abscission and dehiscence zones, a phenomenon that usually occurs in lots of flower tissues.

## Material and methods

Putative transcription factors of the SHINE family were searched in *Eucalyptus* by comparing the *AtSHN* amino acid sequences with the *Eucalyptus* genome assembly (obtained from *Eucalyptus grandis* – http://eucalyptusdb.bi.up.ac.za).

This analysis revealed the existence of two sequences with high similarity to *AtSHN* proteins. Quantitative RT-PCR assays were carried out to verify the expression profile of these genes in different tissues (leaves, flowers and xylem) of the hybrid variety *E. urograndis* (*E. uroplhyla* x *E. grandis*). The RNA extraction was carried out following the protocol described by Zeng & Yang (2002) [3]. The qRT-PCR was performed with the *SYBR® Green PCR Master Mixkit* from *Applied Biosystems*. The Results were analyzed according to the mathematical method described by Pfaffl, 2001 [4].

Plant materials were provided by the International Paper Company, Brazil.

## Results and discussion

**Figure 1:**
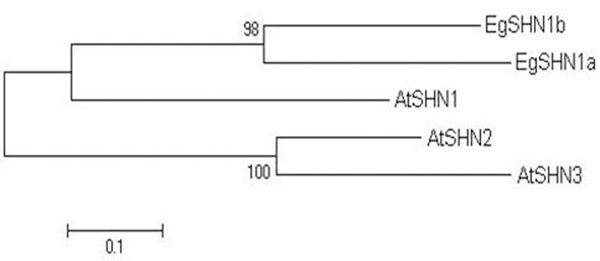
Phylogenetic analysis of SHN genes from *A. thaliana* (*AtSHN1*, *AtSHN2 and AtSHN3*) and the new SHN genes identified in *Eucalyptus grandis* (*EgSHN1a* and *EgSHN1b*). The scale bar of 0,1 corresponds to 10% sequence divergence. Bootstrap values are given for nodes and are considered as value of significance of the branches.

The comparison of the *AtSHN* amino acids sequence with *E. grandis* genome revealed the existence of two putative *SHN* genes in *Eucalyptus* (named *EgSHN1a* and *EgSHN1b*). A phylogenetic analysis showed that these *Eucalyptus* SHN genes are orthologs to the *AtSHN1* of*Arabidopsis thaliana* (Figure 1).

The proteins encoded by *EgSHN1a* and *EgSHN1b* share approximately 58% identity to *AtSHN1*and a conserved gene structure is found between *AtSHN* and both *EgSHN* genes: a single intron is present and located approximately 80 bp from the start codon. In addition, as in *AtSHN1*, *EgSHN* genes contain an AP2 DNA binding domain, which is in accordance to their putative role as transcription factors. More importantly, the domains “mm” and “cm”, which are exclusive of SHN genes, could be identified in the *Eucalyptus* sequences. In the same way that the gene sequence is very correlated between the species, it’s probable that their function are the same two.

**Figure 2:**
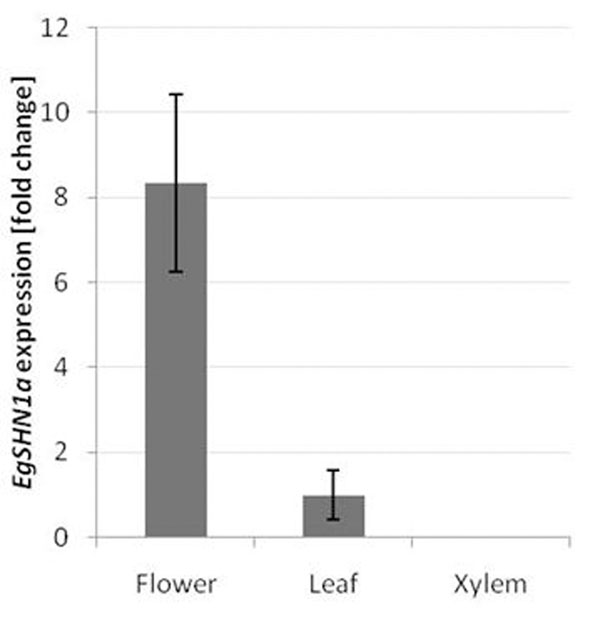
*EgSHN1*a expression ratio in three different tissues: flower (rich in abscission and dehiscence zones), leaf and xylem assessed trough qRT=PCR. Data are expressed as fold change and leaf was chosen as reference condition. Error bars representSE(n=3).

Gene expression analysis revealed a higher expression of *EgSHN1a* in flowers (Figure 2). This result is similar to the *AtSHN1* expression profile [2], suggesting a similar function for *EgSHN1a.*

The expression pattern of the gene *EgSHN1b* is being carried on.

## Conclusions

There are two SHN genes in *Eucalyptus* (*EgSHN1a* and *EgSHN1b*), which are orthologs to *AtSHN1*;

The *EgSHN1a* function might be the same of *AtSHN1* as suggested by their similar expression patterns. It is possible that, in *Eucalyptus*, biosynthesis of cuticle and cell wall in abscission and dehiscence zones is regulated by *EgSHN*’s, as already described in *A. thaliana*[1].

The new genes described here are interesting candidate for the development of transgenic *Eucalyptus*. Overexpression of SHN genes in *A. thaliana* increased tolerance to drought and pathogen attacks and greatly improved cell wall quality.
